# Supplementary material for: Lactucopicrin: A Sesquiterpene Lactone with Anti-Inflammatory Activity Modulates the Crosstalk between NF-kB and AHR Pathways
Source: J Med Chem. 2025 Nov 4;68(21):23406–20. doi: 10.1021/acs.jmedchem.5c02294 (PMC12820960; doi:10.1021/acs.jmedchem.5c02294)
Supplement: Supplementary file 2 [file jm5c02294_si_002.pdf]

Supporting Information

Lactucopicrin: A sesquiterpene lactone with anti-inflammatory activity modulates the crosstalk between NF-kB and AHR pathways

María Ángeles Ávila-Gálvez[a,b], Catarina J G Pinto[b,c,d], Carlos Rafael-Pita[b], Inês P. Silva[b], Aleksandra T. Janowska[f], Sérgio Marinho[c,d], Rory Saitch[f], Yilong Lian[f], Pakavarin Louphrasitthiphol[f], Jonas Protze[g], Gerd Krause[g], Pedro Moura-Alves[c,d,f*], Cláudia Nunes Dos Santos[a,b,e *]

[a] Instituto de Biologia Experimental e Tecnológica (iBET), Av. República, Qta. Marquês, 2780-157 Oeiras, Portugal.

[b] iNOVA4Health, NOVA Medical School|Faculdade de Ciências Médicas, NMS|FCM, Universidade Nova de Lisboa, 1169-056, Lisboa, Portugal.

[c] IBMC, Instituto de Biologia Molecular e Celular, Universidade do Porto, Rua Alfredo Allen, 208, 4200-135, Porto, Portugal.

[d] i3S, Instituto de Investigação e Inovação em Saúde, Universidade do Porto, Rua Alfredo Allen, 208, 4200-135, Porto, Portugal.

[e] NOVA Institute for Medical Systems Biology, NIMSB, Universidade Nova de Lisboa, 1099-085 Lisboa, Portugal

[f] Ludwig Institute for Cancer Research, Nuffield Department of Clinical Medicine, University of Oxford, OX3 7DQ, Oxford, United Kingdom.

[g] FMP, Leibniz-Forschungsinstitut für Molekulare Pharmakologie, 13125 Berlin, Germany.

*Corresponding authors: Cláudia Nunes Dos Santos, claudia.nunes.santos@unl.pt; Pedro Moura-Alves, pmouraalves@i3s.up.pt

Table of Contents

1. Figure S1 .... S2
2. Figure S2 .... S2
3. Figure S3 .... S3
4. Figure S4 .... S3
5. HPLC traces for dihydrolactucin (DHLC) S4
6. HPLC traces for dihydrolactucopicrin (DHLCP) S5
7. HPLC traces for lactucin (LC) S6
8. HPLC traces for lactucopicrin (LCP) S7

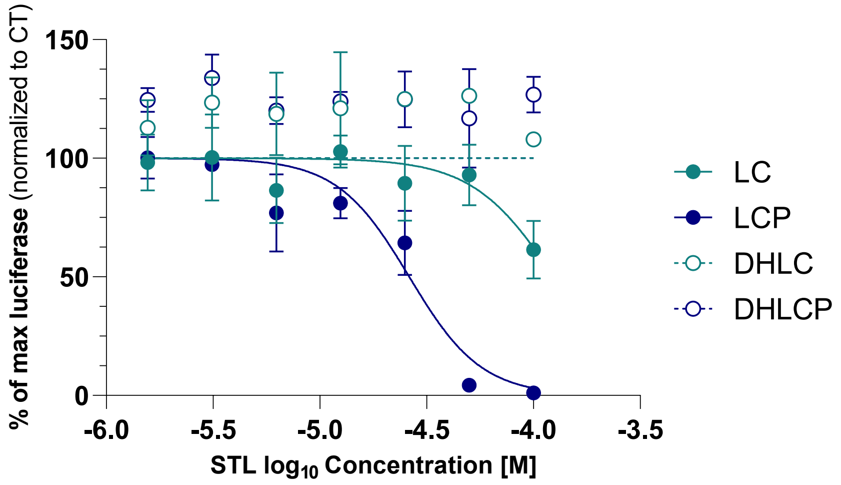

Figure S1. THP-1 NF-kB luciferase reporter cells were exposed for 4h to diverse concentrations of dihydrolactucin (DHLC), dihydrolactucopicrin (DHLCP), lactucin (LC), or lactucopicrin (LCP). Data normalized to CT (DMSO). Data are shown as the mean ± SD.

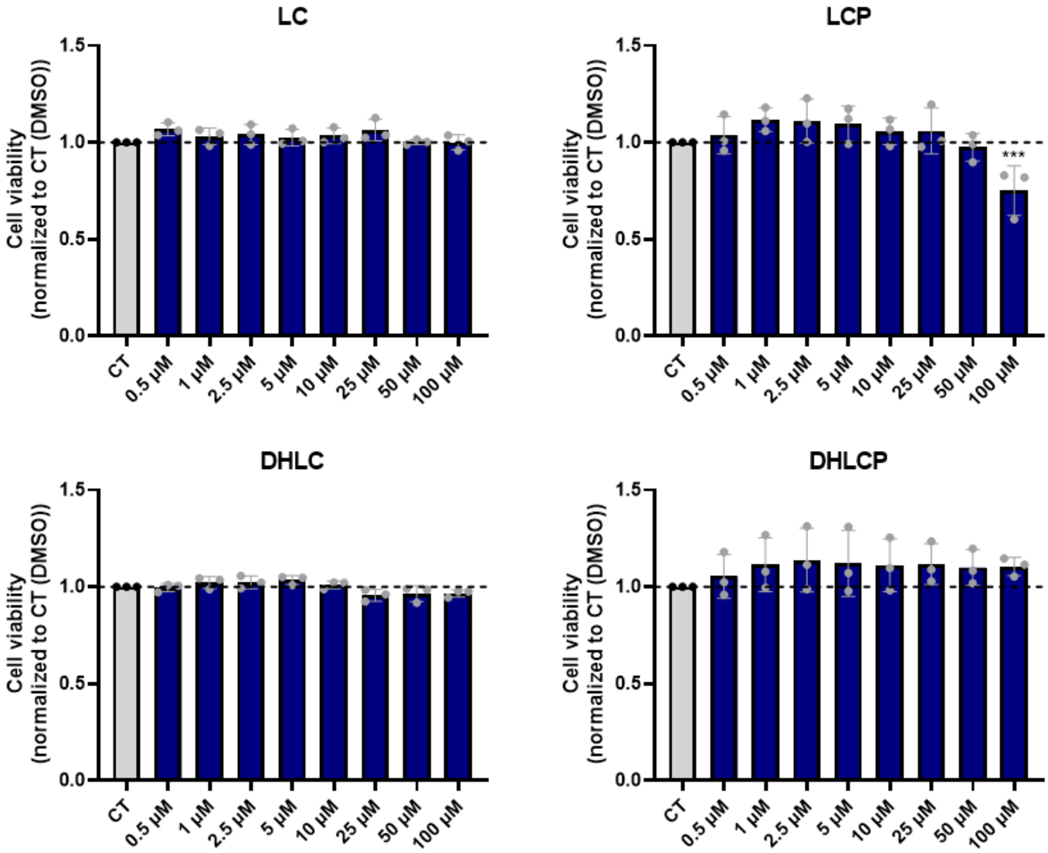

Figure S2. Viability assay in EA.hy926 human endothelial cells. Cells were incubated for 24 hours with different concentrations of each compound or with no addition (control, CT). Cell viability was assessed and is presented as a percentage relative to the control. All values are expressed as mean ± SD, n=3.

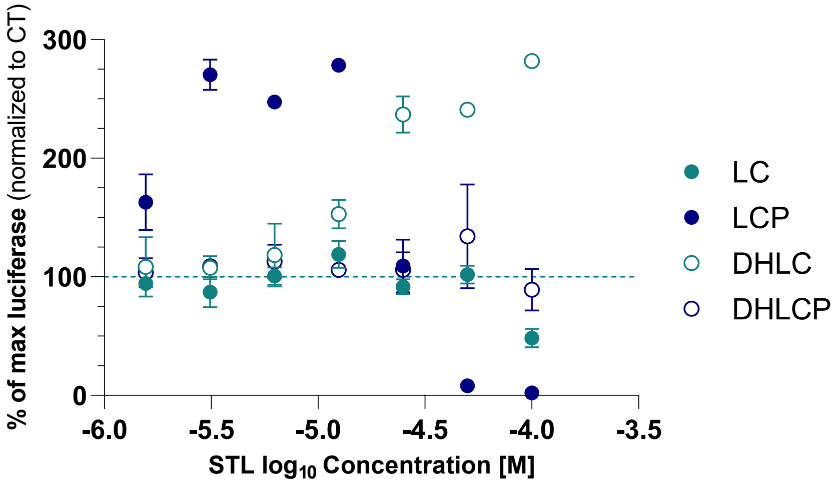

Figure S3. THP-1 AHR luciferase reporter cells were exposed for 4 hours to various concentrations of dihydrolactucin (DHLC), dihydrolactucopicrin (DHLCP), lactucin (LC), or lactucopicrin (LCP). Data normalized to CT (DMSO). Data are shown as the mean ± SD.

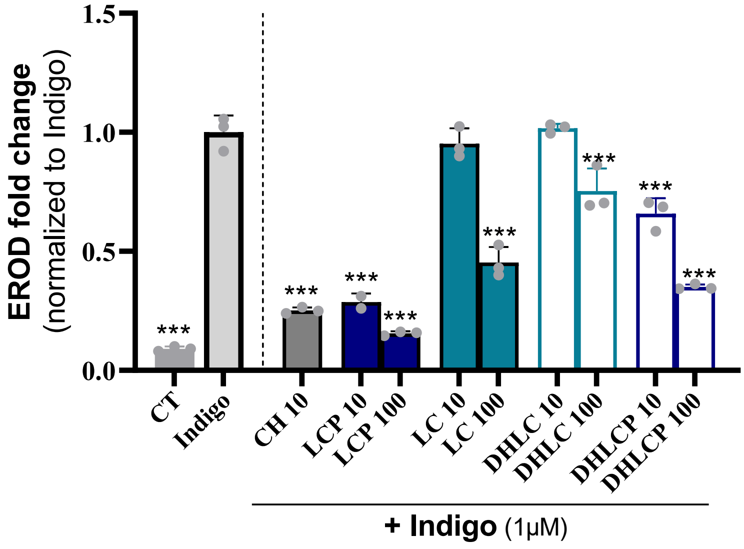

Figure S4. EROD assay in Caco-2 cells exposed for 4h to dihydrolactucin (DHLC), dihydrolactucopicrin (DHLCP), lactucin (LC), lactucopicrin (LCP) (at 10 and 100 μM) or CH223191 (CH) at 10 μM in the presence of Indigo 1 μM. Data shown as means + SD.

DATASHEET

11beta,13-Dihydrolactucin	
Chemical family	SESQUITERPENE
CAS Number	83117-63-9
Empirical formula	$C_{15}H_{18}O_5$
Molecular weight	278,31

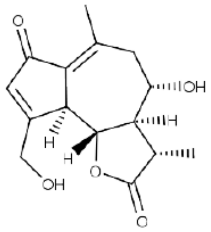

Long term storage	Store at <+8°C<br>Store in dry and dark place.
Shipment conditions	Ambient temperature

Appearance	White to beige coloured Powder
Purity (HPLC)	≥95%

DATASHEET

11beta,13-Dihydrolactucopicrin

Chemical family	SESQUITERPENE
CAS Number	125519-47-3
Empirical formula	$C_{23}H_{24}O_7$
Molecular weight	412,44

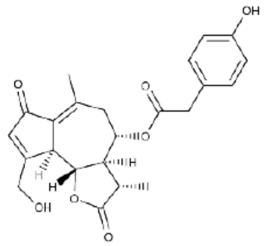

Long term storage	Store at <+8°C<br>Store in dry and dark place.
Shipment conditions	Ambient temperature

Appearance	White to beige coloured Powder
Purity (HPLC)	≥95%

DATASHEET

Lactucin	
Chemical family	SESQUITERPENE
CAS Number	1891-29-8
Empirical formula	$C_{15}H_{16}O_5$
Molecular weight	276,29

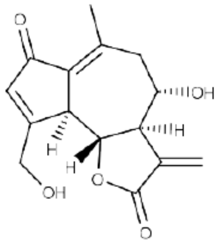

Long term storage	Store at <+8°C
	Store in dry and dark place.
Shipment conditions	Ambient temperature

Appearance	White to beige coloured Powder
Purity (HPLC)	≥95%

DATASHEET

Lactucopicrin

Chemical family	SESQUITERPENE
Synonym	Lactupicrin ; Intybin
CAS Number	65725-11-3
Empirical formula	$C_{23}H_{22}O_7$
Molecular weight	410,43

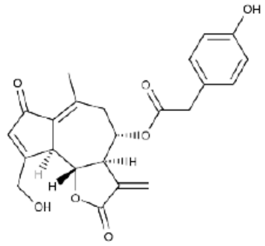

Long term storage	Store at <+8°C<br>Store in dry and dark place.
Shipment conditions	Ambient temperature

Appearance	White to beige coloured Powder
Purity (HPLC)	≥90%
